# Selecting self-and-follower goal-aware leadership styles across sectors: a decision support approach

**DOI:** 10.3389/fpsyg.2026.1685841

**Published:** 2026-04-01

**Authors:** Gergely Czukor, Serhat Yüksel, Serkan Eti, Hasan Dinçer

**Affiliations:** 1 Department of Psychology, Istanbul Bilgi University, Istanbul, Türkiye; 2 School of Business, Istanbul Medipol University, Istanbul, Türkiye; 3 Department of Economics and Management, Khazar University, Baku, Azerbaijan; 4 ARUCAD Research Centre, Arkin University of Creative Arts and Design, Northern Cyprus, Türkiye; 5 University College, Korea University, Seoul, Republic of Korea

**Keywords:** fuzzy decision making, innovation in organizations, leader-follower relationship, leadership assessment, self-and-follower goal-aware leadership, self-goal awareness

## Abstract

Current leadership research falls short of addressing multidimensional performance criteria such as employee engagement, team productivity, innovation, job security, and sustainability in a balanced manner. Instead, it largely focuses solely on follower-focused approaches and fails to systematically assess leaders' own goal awareness. This situation increases the need for holistic and data-driven decision-making models for leadership style selection across various sectors. Studies conducted on this objective are quite limited in the literature. The aim of this study is to determine the most appropriate self-and-follower goal-aware leadership model (SFGAL)-based leadership style for the energy, automotive, healthcare, and information and communication technologies sectors and to reveal differences across sectors. The study evaluates four leadership alternatives (win-win, self-oriented, self-neglecting/over-giving, and lose-lose). Expert weights are determined using a machine learning-based mechanism that considers the demographic and professional characteristics of the experts. Criteria weights are calculated using the criteria importance through intercriteria correlation (CIMAS) method, and the ranking of the alternatives is performed using the combined compromise solution (CoCoSo) technique. The innovative Koch Snowflake fuzzy set approach is used to model uncertainties and linguistic evaluations. The study's key contributions to the literature are as follows: (1) developing a sector-sensitive decision-making framework that enables the generation of unique leadership strategies for different sectors; (2) strengthening uncertainty modeling by applying the Koch Snowflake fuzzy set application within the context of MCDM in leadership assessment; and (3) increasing the reliability and validity of the decision process by employing a demographic and attribute-based approach to expert weighting. Key findings indicate that innovation is the most important criterion in the automotive and information technology sectors, while occupational safety is paramount in the energy sector and employee engagement in the healthcare sector. A win-win leadership style is identified as the most suitable option in all sectors, with self-oriented leadership being the second priority in the energy, healthcare, and information technology sectors.

## 1 Introduction

Leadership has long been recognized as a central mechanism through which organizations influence employee motivation, performance, and wellbeing. As a core organizational and social process, leadership shapes how strategic goals are defined, communicated, and translated into daily work practices, while also influencing how decisions are made and resources are allocated. Through both formal authority and informal influence, leaders create the conditions under which employees interpret their roles, evaluate organizational priorities, and experience their work environment (Burnside et al., 2025). Effective leadership plays a critical role in fostering perceptions of fairness, recognition, and support, which are essential for sustaining positive work attitudes and long-term commitment. Beyond its structural function, leadership is inherently relational, as leaders continuously interact with followers and shape how individual aspirations are aligned with collective organizational objectives. These interactions influence key psychological outcomes such as trust, engagement, psychological safety, and a sense of purpose at work. Leadership practices also affect employees' experiences of autonomy, competence, and relatedness, which are central drivers of motivation and resilience, particularly in demanding or uncertain contexts (Le and Gia, 2025). In contemporary organizational environments characterized by rapid change, technological disruption, and increasing performance pressures, leaders are expected to balance multiple and often competing demands, including productivity, innovation, employee wellbeing, and sustainability. Consequently, leadership is widely regarded as a key determinant of organizational effectiveness, as it serves as a primary lens through which organizations can understand and address challenges related to declining engagement, weakened trust, and reduced employee wellbeing.

The global outlook on the desired outcomes of leadership shows a pessimistic picture: only 27% of employees globally are engaged with their work, 29% trust their leaders, and 21% feel that their leaders support their growth (DDI, 2025; Gallup, 2025). These disappointing statistics show an engagement crisis and the absence of transformational and authentic leadership in organizations. These negative employee experiences suggest that scholars and practitioners should revisit leadership theories and their applications. While the general leadership literature is follower-centric, the applied organizational implementation often fails due to limitations in addressing leader-follower relationship barriers that prevent engagement. Self-and-follower goal-aware leadership model (SFGAL) enhances employee engagement in organizations, and to contribute to leadership theory. SFGAL focuses on the interplay of two dimensions, the leaders' awareness of their followers' goals, and the leaders' self-awareness of their own goals. SFGAL emerges from the dyadic tradition in leader-member exchange (LMX). However, it extends the scope from quality of exchanges to specific work-related and professional gains within a self-deterministic perspective (Yang et al., 2025). The model expands the literature on leaders' self-awareness of their emotions and values to work and professional goal-directed awareness. Concurrently, it contributes to follower-centric approaches, emphasizing that the leaders' self-goal awareness is equally important to their awareness of the followers' needs. The proposed model aims to provide explanatory insight into failing leader-follower relations and actionable insight to improve those relationships (Masenya and Ngoepe, 2025). SFGAL provides a theoretical, assessment, and development perspective to elaborate, assess, and nurture the extent to which leaders generate mutual gains by attending to (a) their professional needs/aspirations (e.g., stress reduction, career growth) and (b) their followers' needs (e.g., skill development, role satisfaction).

Despite the extensive body of leadership research, existing theories and models tend to adopt a predominantly one-sided perspective by focusing primarily on leaders' responsibility to attend to followers' needs, motivation, and development. Approaches such as transformational, authentic, servant, and leader–member exchange leadership have made substantial contributions by demonstrating how follower-oriented behaviors enhance engagement, commitment, and performance. However, these frameworks often implicitly frame self-focused leader behaviors as undesirable, self-interested, or even harmful, resulting in limited systematic attention to leaders' own work-related goals, aspirations, and wellbeing. This conceptual imbalance creates a critical gap in understanding how leaders navigate their dual role as both organizational agents with personal professional objectives and relational actors responsible for supporting followers. In complex organizational environments characterized by competing performance demands, uncertainty, and increasing accountability, leaders are required to simultaneously manage their own stress, career development, and effectiveness while also fostering positive outcomes for their teams. Existing leadership theories provide insufficient guidance for explaining how these dual demands can be balanced, or why leader–follower relationships sometimes deteriorate despite strong follower-oriented intentions. As a result, leadership practice often struggles to account for situations in which excessive self-sacrifice leads to leader burnout or, conversely, where strong self-orientation undermines trust and engagement. Addressing this gap requires a framework that explicitly integrates leaders' self-goal awareness with their awareness of followers' goals, thereby offering a more holistic understanding of leadership effectiveness. It is within this context that the Self-and-Follower Goal-Aware Leadership model is proposed as a response to the limitations of existing approaches.

The SFGAL model addresses two limitations in leadership research and development: first, SFGAL provides an amendment concerning the imbalance of favoring follower-focused leadership (e.g., transformational leadership), while largely disapproving of self-focused leadership behaviors as self-interested. This new angle on this division emphasizes the leaders' focus on their self-goals besides helping followers to achieve their goals, ensuring mutual gains in the leader-follower relationship (Burnside et al., 2025). Thus, SFGAL embraces the strengths of existing leadership approaches, i.e., their focus on the leaders' role to attend to their followers' needs. It also improves limitations concerning leaders' awareness of their self-goals. Second, SFGAL addresses the restricted measurement application of self-determination theory–based psychological needs as specific work-related constructs. Leadership theories commonly propose that effective leaders attend their followers' work-related/professional needs and aspirations (Burns, 1978). However, the current self-determination theory-based measures have practical limitations for overwhelmingly focusing on psychology, rather than specific work-related outcomes, such as employee needs for necessary tools, fair compensation, input in to work decisions, to list a few. The proposed SFGAL measure focuses on specific work and profession-based competence, autonomy, and relatedness gains resulting from the leaders' leadership engagement (Schermuly et al., 2025).

In the leadership literature, existing studies predominantly emphasize follower-centered approaches that focus on meeting employees' needs, motivation, and development, while giving limited systematic attention to leaders' own goal awareness and professional aspirations. Although this body of research has substantially advanced understanding of how leadership influences employee outcomes, it has also produced a conceptual imbalance by implicitly portraying self-focused leader behaviors as undesirable or incompatible with effective leadership. This limitation becomes particularly salient in contemporary organizational contexts, where leaders are required to manage multiple and often competing performance dimensions, such as employee commitment, productivity, innovation, job security, and sustainability. Addressing these multidimensional challenges requires leadership frameworks that move beyond one-sided perspectives and explicitly consider how leaders balance their own goals with those of their followers across different organizational and sectoral settings.

In parallel, organizational and leadership research has increasingly recognized the value of analytical and decision-support approaches for examining complex managerial phenomena. Multi-criteria perspectives provide a structured foundation for evaluating leadership effectiveness when outcomes depend on trade-offs among multiple organizational priorities rather than on single performance indicators. Moreover, advances in uncertainty modeling have highlighted the importance of capturing the ambiguity and subjectivity inherent in expert judgments and linguistic assessments, particularly in leadership evaluation contexts. Despite their demonstrated value in other fields, such approaches remain underutilized in leadership research, limiting the ability to conduct systematic, sector-sensitive assessments of leadership styles. Against this background, the purpose of the present study is to develop and apply a structured decision-support framework that enables the comparative evaluation of Self-and-Follower Goal-Aware Leadership styles across different sectors, while accounting for multidimensional performance criteria and sector-specific priorities.

The background information regarding the topic is denoted in the second section. The third part focuses on the details of the proposed model. In the following part, the results of this model are given. The final parts give information about the discussion and conclusion.

## 2 Background

### 2.1 Follower-focused leader behaviors

The prominent leadership theories, including transformational, charismatic, authentic, and LMX, commonly propose that leadership entails attending to followers' needs (Burns, 1978). The leadership influence process occurs as the leader communicates a vision and collective goals aligned with their followers' interests. Although the primary aim is to benefit the organization by motivating the followers to achieve organizational goals, the theories indicate that followers are motivated when their needs and goals are addressed by their leaders (Oppong and Oduro-Asabere, 2025). Transformational leadership theory proposes idealized influence, individualized consideration, inspirational motivation, and intellectual stimulation to motivate followers to achieve valuable objectives and show outstanding performance. These leadership behaviors satisfy followers' psychological needs of competence, autonomy, and relatedness, enhancing specific work outcomes, such as increased self-efficacy, satisfaction, and commitment (Kovjanic et al., 2012). Authentic leadership theory encourages leaders to develop self-awareness, relational transparency, balanced processing, and a strong moral code to motivate followers. These aspects of authentic leadership enhance followers' self-actualization via moral content and the satisfaction of basic needs (Günzel-Jensen et al., 2024). Likewise, charismatic leaders attend to their followers' competence needs by proposing high expectations while expressing trust in their followers' capabilities (Eden, 1990). In sum, the leadership literature explicitly argues and demonstrates that leaders maintain awareness of their followers' needs and attend to them to be effective. When leaders do so, their followers show higher commitment, satisfaction, and performance.

### 2.2 Self-goal-focused leader behaviors

The literature provides an imbalanced view of follower-focused and self-focused leader behaviors, the former being desirable and the latter non-desirable. Self-oriented leader behaviors, such as pseudo-transformational leadership, are mainly studied in destructive leadership (Barling et al., 2008). In those, the leaders are motivated by their self-interest to generate gains for themselves, but intentionally or unintentionally ignore the interests of the followers or the organization (Okpala, 2023; Padilla et al., 2007). However, self-oriented behaviors may not necessarily indicate self-interested leadership; on the contrary, those behaviors may help leaders to be more effective. Leaders have various legitimate work-related basic and growth level needs, such as maintaining task concentration, managing stress, boosting self-development, achieving high performance, attaining promotion, being able to communicate well, being competent, and networking in the organization, to name a few (Correa et al., 2025). A lack of self-goal orientation can have negative consequences for leaders. Research on the dark side of transformational leadership draws attention to leaders' exhaustion and burnout from heavily attending to their followers' needs, while ignoring those of their own (Lin et al., 2019). Further, followers dislike leaders who chronically sacrifice their goals, as it can imply a lack of self-respect and competence, violating expectations of reciprocity (Blau, 1964). Additionally, leader self-neglect may result in low agency attributions (Kelley, 1973; McLeod, 2020), and perceptions of limited authenticity as self-and others-awareness are deemed important for perceived authenticity (Gardner et al., 2005). Therefore, the literature should embrace constructive self-focused leader behaviors as a leadership asset to maintain effectiveness.

### 2.3 SFGAL model

The conceptual background of SFGAL entails the interplay between the leader's awareness of (a) his/her needs and aspirations, and (b) those of his/her followers. The model proposes a two-dimensional grid for empirical testing, involving four leadership types across these two types of awareness. The model methodologically is built on the Blake & Mouton Leadership Grid, which outlines four leadership styles based on the interaction between leaders' focus on production vs. people in a two-dimensional grid. SFGAL retains follower orientation; however, it will refer to the leaders' tendency to generate work-related and professional gains for the followers. Further, the production axis from the prior model represents the leaders' awareness of their self-goals to generate gains for themselves (Le and Gia, 2025). Building on previous two-dimensional models, SFGAL proposes four leadership style quadrants for hypothesis testing, emerging from the interplay between the leaders' self-goal and follower-goal awareness (van Knippenberg et al., 2025). Hypothetically, these styles emerge from the relative ratio of leaders' self-goal awareness and their awareness of their followers' goals. The proposed styles are *win-win* (high self and follower focus), *self-oriented* (high self and low follower focus), *self-neglecting/over-giving* (low self and high follower focus), and *lose-lose* (low self and low follower focus). The study aims to empirically validate these styles, supported by prominent leadership theory and research.

### 2.4 Proposed SFGAL leadership styles

#### 2.4.1 Win-win leaders: high self-focus, high follower-focus

SFGAL proposes that the most desirable leadership style corresponds with the win-win leaders, who display high self and follower-focused behaviors. These leaders attend to their own needs and those of their followers, and their leadership generates gains for themselves as leaders and for their followers (Rehan et al., 2025). Although leadership theories do not explicitly address leader needs and gains, they implicitly indicate that the leaders profit from the leader-follower relationship while also attending to their followers' needs. For example, transformational leaders propose goals and visions aligned with the employees' values and professional aspirations (Avolio and Gardner, 2005). The followers internalize the leaders' goals and benefit the leader by showing proactive behaviors to pursue them (Klug et al., 2018). Further, high leader-member exchange (LMX) involves reciprocity, to which the followers respond by exerting effort to help their leader (Ilies et al., 2007). In such ideal forms of leadership, leaders exert effort to learn and act upon their followers' needs, hence the followers accumulate valuable gains. When the leaders attend to the followers' needs and aspirations, the organization shares the gains: productivity increases when leader-follower goals are aligned (Klug et al., 2018), and turnover intention drops when leaders reduce stressors (Nielsen et al., 2023). In short, win-win leaders benefit themselves, their followers, and the organization. Potential dark side of win-win leaders may include groupthink deriving from the enhanced collective enthusiasm and social identification with the group.

#### 2.4.2 Self-neglecting/over-giving leadership: low self-focus, high follower-focus

The two-dimensional aspect of the SFGAL model allows for forecasting leadership cases in which the leaders are strongly dedicated to their followers' development but lack self-goal awareness. The self-neglecting/over-giving leadership style refers to leaders who are high in follower-focused, but low on self-focused goal-awareness. These leaders are perceived to attend to their followers' needs and help them attain their aspirations, but they lack self-goal awareness; hence, they appear self-neglecting (Tho et al., 2025). Self-neglecting/over-giving leaders chronically disregard their goals and wellbeing as a habitual behavior in their effort to support followers, undermining their authority and effectiveness. Hence, self-neglecting/over-giving leadership does not refer to situational heroic acts, when leaders demonstrate self-sacrifice for a higher cause. Self-neglecting/over-giving leadership may mistakenly seem favorable based on the leadership literature's positive approach toward follower-focused and negative approach toward self-focused leadership behaviors (van Knippenberg et al., 2025). The same study indicates that female leaders more commonly experience such exhaustion under the pressure of widespread stereotypes about women being nurturing: they exert imbalanced effort to develop their followers via individualized consideration and inspirational motivation, relative to how much these leaders focus on their needs and aspirations. Failing to maintain self-goal awareness and showing signs of self-neglect can negatively affect how followers perceive their leaders.

Accordingly, leaders should convey self-focused behaviors to maintain leadership effectiveness. Such self-focus should be perceived positively by the followers, especially when aligned with the leader's awareness of the followers' goals as outlined in win-win leadership. However, collectivist cultures may be more tolerant of self-neglecting/over-giving leadership when followers expect the leaders to prioritize collective welfare, and when such self-sacrifice indicates moral authority, as opposed to individualistic cultures that are more likely to consider self-neglect as a weakness. Furthermore, female leaders in societies with rigid gender roles may particularly emphasize collective goals and understate their self-goals. Therefore, the application of SFGAL in different cultures may require adjustments.

#### 2.4.3 Self-oriented leader: high self-focus, low follower-focus

Self-oriented *leaders* have high self-focus and low follower-focus. This leadership style portrays leaders who actively attend to their needs and pursue their aspirations, but intentionally or unintentionally fail to attend to their followers' needs. The literature warns against self-interested leaders whose behaviors can harm the organization (Mackey et al., 2020; Padilla et al., 2007). Self-oriented leadership is generally associated with destructive leaders with personality-related inclinations for egoistic behaviors, such as in the case of chronic narcissism. In general, followers who attribute self-interest to their leaders indicate negative emotions and reduced OCB (Chernyak-Hai and Tziner, 2021).

#### 2.4.4 Lose-lose leadership: low self-focus, low follower-focus

Lose-lose leadership style describes managers who show no awareness of their own and their followers' needs and provide no self- and follower gains through their leadership. These leaders may be unmotivated, unknowledgeable, incapable of focusing on their individually based goals, or attending to their followers' needs. Thus, this leadership style fails to generate mutual gains in the leader-follower relationship. The workplace alienation literature provides insight into employees being disengaged and negative about their work. When leaders feel alienated, followers and the organization may face detrimental consequences (Babin and Boles, 1996). Alienated managers are emotionally disengaged, hence they are indifferent toward their growth potential. Additionally, they show no concern for their followers' needs, resulting in them disengaging, performing poorly (Khan et al., 2019), and showing enhanced turnover intention (Vanderstukken and Caniëls, 2021).

## 3 Methodology

**Figure 1:**
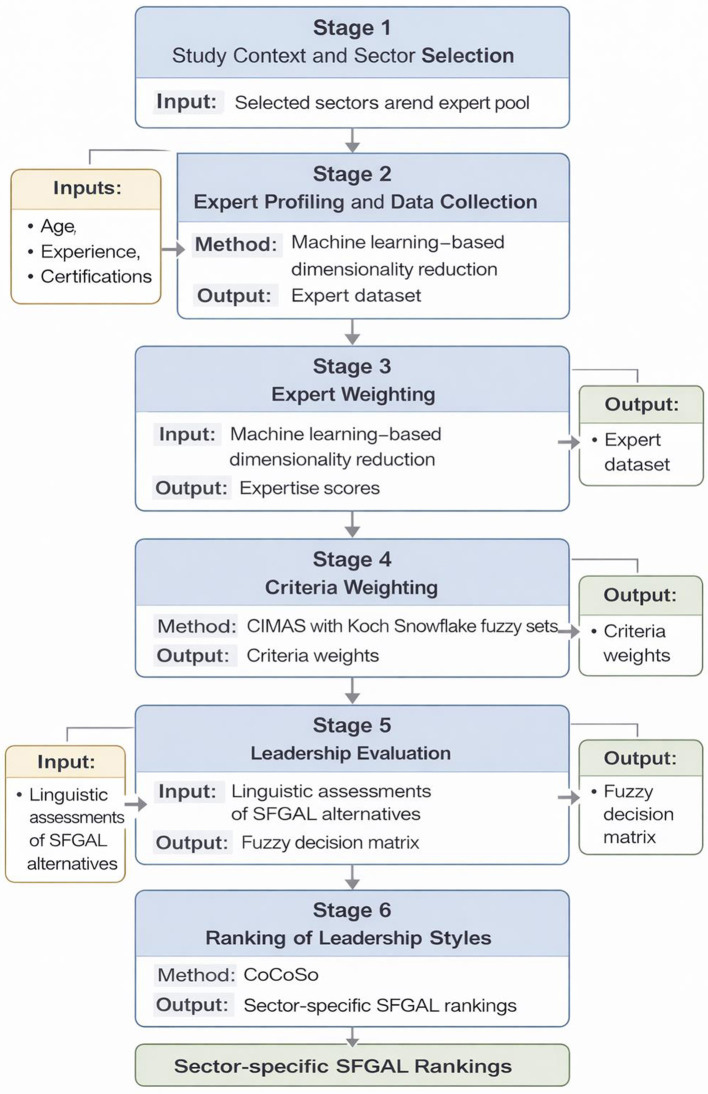
Workflow of the proposed decision-support framework for SFGAL-based leadership selection.

This manuscript, which explores the selection of the most appropriate leadership style based on Self-and-Follower Goal-Aware Leadership (SFGAL) for various sectors, utilizes a fuzzy decision model. This fuzzy decision model incorporates expert prioritization, criterion weighting, and leadership ranking methods. In analyses conducted for four different sectors, fuzzy set theory is utilized to measure the uncertainty arising from linguistic expressions. In the fuzzy decision model, where a machine learning algorithm is used for expert prioritization, CIMAS is preferred for calculating criterion weights. The CoCoSo method is integrated into the model for ranking leaderships. The stages of the fuzzy decision model are presented in Figure 1.

Figure 1 presents the overall workflow of the proposed decision-support framework. The process begins with the identification of the study context and the selection of sector-specific experts, whose demographic and professional characteristics constitute the initial input. In the next stage, machine learning-based dimensionality reduction is applied to derive expertise scores, which are subsequently used to weight expert evaluations. The framework then proceeds to the weighting of leadership criteria using the CIMAS method, incorporating expert assessments under uncertainty modeled by Koch Snowflake fuzzy sets. Finally, the weighted criteria and fuzzy evaluations of leadership alternatives serve as inputs to the CoCoSo method, which produces the final ranking of SFGAL leadership styles for each sector. This structured workflow clarifies the logical sequence of analytical steps and the interdependencies between model components.

This study adopts an integrative methodological perspective in which leadership theory, expert-based evaluation, uncertainty modeling, and multi-criteria decision support are combined within a single analytical framework. Rather than applying these techniques as independent or sequential tools, the proposed design treats their integration as a core methodological contribution. This approach enables the simultaneous consideration of leaders' self-goal awareness, follower-goal awareness, and sector-specific performance priorities, while systematically accounting for expert heterogeneity and uncertainty in judgments. By embedding advanced decision-support techniques within a theoretically grounded leadership framework, the methodology moves beyond conventional applications of multi-criteria analysis and offers a structured means of operationalizing complex leadership constructs in applied organizational settings.

### 3.1 Study design and data collection

This study adopts a structured expert-based decision-support research design to evaluate Self-and-Follower Goal-Aware Leadership (SFGAL) styles across multiple sectors. The research is conducted within four key industries that exhibit distinct organizational dynamics and leadership demands: energy, automotive, healthcare, and information and communication technologies. These sectors were selected to ensure variability in performance priorities, regulatory environments, and organizational complexity, thereby enabling a comparative and sector-sensitive leadership assessment.

The empirical inputs of the study are derived from an expert panel composed of senior professionals with extensive experience in human resources management, organizational leadership, and strategic decision-making. A purposive sampling strategy was employed to ensure that participants possessed both sector-specific expertise and long-term professional experience relevant to leadership evaluation. In total, 10 experts participated in the study, each with a minimum of 25 years of professional experience. All experts held senior or executive-level positions in their respective sectors, such as human resources directors, senior managers, or organizational consultants, and possessed at least a university-level degree, with several holding postgraduate qualifications.

Data were collected through structured online interviews and evaluation forms. During this process, experts were asked to assess leadership criteria and SFGAL leadership alternatives using predefined linguistic scales. These assessments formed the basis for the decision matrices used in the analysis. In addition to leadership evaluations, demographic and professional information, including age, years of experience, and number of certifications or patents, was collected to inform the expert weighting process. This design ensures transparency in data collection and allows for the systematic integration of expert judgment into the proposed decision-support framework.

### 3.2 Machine learning for expert prioritization

Machine learning has recently been frequently used in data science to calculate parameters. One of the machine learning techniques developed for various purposes is dimensionality reduction algorithms used to generate latent structures. Because direct measurement of expert expertise is not possible, it must be obtained using related variables. One of the most fundamental criticisms in the literature is that every expert is evaluated equally (Chen et al., 2025). Despite this criticism, the manuscript aims to obtain expert expertise scores through dimensionality reduction. The process for obtaining expertise scores through dimensionality reduction is described below.

In the first stage of the fuzzy decision model, observed variables are collected to prioritize experts. In other words, variables related to expertise, such as years of experience and number of projects managed, are obtained to create the data set formed in Equation 1.


$$\begin{array}{l} P=\left[\begin{array}{ccc} p_{11} & \cdots & p_{1y}\\ \vdots & \ddots & \vdots \\ p_{d1} & \cdots & p_{dy}\; \end{array}\right] \end{array}$$ (1)

Wherein d and y are the numbers of experts and variables related to expertise, respectively. However, the unit variables of the columns in this data set are different. In other words, one column might contain year information while the other might contain amount information. Therefore, the values need to be standardized. For this purpose, the values are centralized with Equations 2, 3 and then made unitless with Equation 4.


$$\begin{array}{lll} {\bar{p}}_{j} & = & \frac{1}{d}\overset{d}{\underset{i=1}{\sum }}p_{ij} \end{array}$$ (2)

$$\begin{array}{lll} c_{ij} & = & p_{ij}-{\bar{p}}_{j} \end{array}$$ (3)

$$\begin{array}{lll} r_{ij} & = & \frac{c_{ij}}{\sqrt{\sum_{i=1}^{d}c_{ij}^{2}}} \end{array}$$ (4)

Thus, a standardized matrix is obtained that accepts the standardized values obtained through this process as elements. The standardized matrix is shown in Equation 5.


$$\begin{array}{l} R=\left[\begin{array}{ccc} r_{11} & \cdots & r_{1y}\\ \vdots & \ddots & \vdots \\ r_{d1} & \cdots & r_{dy} \end{array}\right] \end{array}$$ (5)

Then, the covariance coefficients between columns of standardized matrix are calculated with Equation 6.


$$\begin{array}{l} cov_{ij}=\frac{\sum_{t=1}^{d}\left(r_{ti}-{\bar{r}}_{i}\right)\left(r_{tj}-{\bar{r}}_{j}\right)}{d} \end{array}$$ (6)

The covariance matrix that accepts the covariance coefficients as elements is constructed. This matrix is formed as Equation 7.


$$\begin{array}{l} A=\left[\begin{array}{ccc} cov_{11} & \cdots & cov_{1y}\\ \vdots & \ddots & \vdots \\ cov_{y1} & \cdots & cov_{\mathrm{yy}} \end{array}\right] \end{array}$$ (7)

Afterwards, the eigenvalues of the covariance matrix are estimated by solving Equation 8. The solution set of this equation has y elements.


$$\begin{array}{l} \det\left(A-\theta \right)=0 \end{array}$$ (8)

Wherein I is a y × y dimensional identity matrix. Next, the maximum value of θ is selected for maximum variance or minimum loss knowledge. The eigenvector corresponding to this maximum eigenvalue is obtained by solving Equation 9.


$$\begin{array}{l} \left(A-\theta^{max}\right)\nu =0 \end{array}$$ (9)

Finally, the projection of data set is created using Equation 10. Then, the projection values are normalized with Equation 11.


$$\begin{array}{lll} h_{i} & = & \overset{y}{\underset{j=1}{\sum }}p_{ij}\nu_{j} \end{array}$$ (10)

$$\begin{array}{lll} {𝔭}_{i} & = & \frac{h_{i}}{\sum_{i\;=}^{d}h_{i}} \end{array}$$ (11)

Wherein 𝔭 is the expertise score of experts and used in the computation steps of CIMAS and CoCoSo.

### 3.3 Koch snowflake fuzzy sets

Fuzzy sets are a unique theory that allows for word processing. By incorporating the uncertainty inherent in linguistic expressions into the analysis, fuzzy sets provide useful tools for obtaining more realistic and accurate results. Koch Snowflake fuzzy sets are among the most widely used fuzzy sets in the literature. These fuzzy sets are inspired by fractal geometry. This set is unique in that it accurately reflects the boundless framework and limited meaning of linguistic expressions (Joshua and Balasubramaniam, 2025). A Koch Snowflake fuzzy set (k) is defined as in Equation 12, where d is a universe of discourse.


$$\begin{array}{l} k=\left\{\langle x,\alpha_{k}\left(x\right),\beta_{k}\left(x\right)\rangle |x\in d.\right\} \end{array}$$ (12)

Wherein α_k_ and β_k_ are the membership and non-membership functions. These functions are satisfied the condition in Equation 13.


$$\begin{array}{l} 0\leq \alpha_{k}^{1.26}+\beta_{k}^{1.26}\leq 1 \end{array}$$ (13)

Wherein, 1.26 comes from the dimension of the Koch Snowflake fractal, which is log(4)/log(3). Let $\tilde{F}$ and $\tilde{G}$ be two Koch Snowflake fuzzy numbers. Some basic mathematical operators are identified by Equations 14–18.


$$\begin{array}{lll} \tilde{F}⊕\tilde{G} & = & \left(\begin{array}{c} \sqrt[{1.26}]{\frac{\alpha_{\tilde{F}}^{1.26}+\alpha_{\tilde{G}}^{1.26}-\alpha_{\tilde{F}}^{1.26}\alpha_{\tilde{G}}^{1.26}-\left(1-\xi \right)\alpha_{\tilde{F}}^{1.26}\alpha_{\tilde{G}}^{1.26}}{1-\left(1-\xi \right)\alpha_{\tilde{F}}^{1.26}\alpha_{\tilde{G}}^{1.26}}},\\ \frac{\beta_{\tilde{F}}\beta_{\tilde{G}}}{\sqrt[{1.26}]{\xi +\left(1-\xi \right)\left(\beta_{\tilde{F}}^{1.26}+\beta_{\tilde{G}}^{1.26}-\beta_{\tilde{F}}^{1.26}\beta_{\tilde{G}}^{1.26}\right)}} \end{array}\right) \end{array}$$ (14)

$$\begin{array}{lll} \tilde{F}⊗\tilde{G} & = & \left(\begin{array}{c} \frac{\alpha_{\tilde{F}}\alpha_{\tilde{G}}}{\sqrt[{1.26}]{\xi +\left(1-\xi \right)\left(\alpha_{\tilde{F}}^{1.26}+\alpha_{\tilde{G}}^{1.26}-\alpha_{\tilde{F}}^{1.26}\alpha_{\tilde{G}}^{1.26}\right)}},\\ \sqrt[{1.26}]{\frac{\beta_{\tilde{F}}^{1.26}+\beta_{\tilde{G}}^{1.26}-\beta_{\tilde{F}}^{1.26}\beta_{\tilde{G}}^{1.26}-\left(1-\xi \right)\beta_{\tilde{F}}^{1.26}\beta_{\tilde{G}}^{1.26}}{1-\left(1-\xi \right)\beta_{\tilde{F}}^{1.26}\beta_{\tilde{G}}^{1.26}}} \end{array}\right) \end{array}$$ (15)

$$\begin{array}{lll} ℸ⊙\tilde{F} & = & \left(\begin{array}{c} \sqrt[{1.26}]{\frac{{\left[1+\left(\xi -1\right)\alpha_{\tilde{F}}^{1.26}\right]}^{ℸ}-{\left(1-\alpha_{\tilde{F}}^{1.26}\right)}^{ℸ}}{{\left[1+\left(\xi -1\right)\alpha_{\tilde{F}}^{1.26}\right]}^{ℸ}-\left(\xi -1\right){\left(1-\alpha_{\tilde{F}}^{1.26}\right)}^{ℸ}}},\\ \frac{\sqrt[{1.26}]{\xi }\beta_{\tilde{F}}^{ℸ}}{\sqrt[{1.26}]{{\left[1+\left(\xi -1\right)\left(1-\beta_{\tilde{F}}^{1.26}\right)\right]}^{ℸ}+\left(\xi -1\right)\beta_{\tilde{F}}^{ℸ1.26}}} \end{array}\right) \end{array}$$ (16)

$$\begin{array}{lll} {\tilde{F}}^{ℸ} & = & \left(\begin{array}{c} \frac{\sqrt[{1.26}]{\xi }\alpha_{\tilde{F}}^{ℸ}}{\sqrt[{1.26}]{{\left[1+\left(\xi -1\right)\left(1-\alpha_{\tilde{F}}^{1.26}\right)\right]}^{ℸ}+\left(\xi -1\right)\alpha_{\tilde{F}}^{ℸ1.26}}},\\ \sqrt[{d}]{\frac{{\left[1+\left(\xi -1\right)\beta_{\tilde{F}}^{1.26}\right]}^{ℸ}-{\left(1-\beta_{\tilde{F}}^{1.26}\right)}^{ℸ}}{{\left[1+\left(\xi -1\right)\beta_{\tilde{F}}^{1.26}\right]}^{ℸ}-\left(\xi -1\right){\left(1-\beta_{\tilde{F}}^{1.26}\right)}^{ℸ}}} \end{array}\right)\; \end{array}$$ (17)

$$\begin{array}{lll} {\tilde{F}}^{C} & = & \left(\beta_{\tilde{F}},\alpha_{\tilde{F}}\right)\; \end{array}$$ (18)

Wherein ℸ and ξ are non-negative values. In addition, the score and accuracy functions are calculated with the help of Equations 19, 20, respectively.


$$\begin{array}{lll} score\left(\tilde{F}\right) & = & \alpha^{1.26}-\beta^{1.26} \end{array}$$ (19)

$$\begin{array}{lll} acc\left(\tilde{F}\right) & = & \alpha^{1.26}+\beta^{1.26} \end{array}$$ (20)

### 3.4 CIMAS

The CIMAS method is a current method used to determine the importance of criteria and is one of the few methods that incorporates expert experience into the analysis. However, the traditional CIMAS method uses years of experience as an indicator of expert experience, which produces narrow results. In this manuscript, expert expertise scores derived from machine learning are used as the experience indicator. Furthermore, the computational process of the extended CIMAS method integrated with Koch Snowflake fuzzy numbers is detailed below (Yalçin et al., 2025).

Firstly, the criteria set is defined. Experts rate the importance of criteria using linguistic terms. The assessments are transformed into Koch Snowflake fuzzy numbers. Then, the fuzzy input data matrix is created as Equation 21.


$$\begin{array}{l} F=\left[\begin{array}{ccc} {𝔣}_{11} & \cdots & {𝔣}_{1n}\\ \vdots & \ddots & \vdots \\ {𝔣}_{d1} & \cdots & {𝔣}_{dn} \end{array}\right] \end{array}$$ (21)

Wherein *n* is the number of criteria. Then, the elements of this matrix are normalized using Equations 22, 23.


$$\begin{array}{lll} {𝔶}_{ij} & = & {𝔣}_{ij}\;for\;useful \end{array}$$ (22)

$$\begin{array}{lll} {𝔶}_{ij} & = & {{𝔣}_{ij}\;}^{C}for\;useless\; \end{array}$$ (23)

Afterwards, the normalized elements are multiplied by the expertise scores of experts with Equation 24.


$$\begin{array}{l} z_{ij}={𝔶}_{ij}{𝔭}_{i} \end{array}$$ (24)

Wherein the multiplication is identified in Equation 16. Next, the multiplied elements are defuzzified by Equation 25.


$$\begin{array}{l} \gamma_{ij}=score\left({\text{z}}_{ij}\right) \end{array}$$ (25)

The maximum and minimum elements of defuzzified elements are obtained via Equations 26, 27.


$$\begin{array}{lll} {max}_{j} & = & \underset{i}{\max}\gamma_{ij} \end{array}$$ (26)

$$\begin{array}{lll} {min}_{j} & = & \underset{i}{\max}\gamma_{ij} \end{array}$$ (27)

The difference between the maximum and minimum elements are computed using Equation 28.


$$\begin{array}{l} \Delta_{j}=max_{j}-min_{j} \end{array}$$ (28)

In the last step, the weights of criteria are determined with Equation 29.


$$\begin{array}{l} \omega_{j}=\frac{\Delta_{j}}{\sum_{j=\;1}^{n}\Delta_{j}} \end{array}$$ (29)

Finally, the reliability index is tested. For this, the second assessments are collected with percentages from each expert. The average of the second assessments is obtained for each criterion and RI value is calculated by Equation 30.


$$\begin{array}{l} RI=\frac{\sum_{j=1}^{n}|100\omega_{j}-A_{j}|}{100} \end{array}$$ (30)

Wherein *A*_*j*_ is the average of the second assessment for *j*^*th*^ criterion. The RI value must be smaller than 0.1 for reliability.

### 3.5 CoCoSo

CoCoSo is used to rank alternatives. CoCoSo ranks alternatives by considering three different strategies. This feature is one of the advantages of the method. Furthermore, the computational process of the CoCoSo method integrated with Koch Snowflake fuzzy numbers is introduced below (Tarafdar et al., 2025).

After defining the criteria, the alternatives are identified. Then, the experts evaluate the alternatives according to criteria. The evaluations are transformed into Koch Snowflake fuzzy numbers.

For each criterion, each expert's alternative evaluation is multiplied by the expert's expertise score using Equation 16. These expert weighted values for each criterion are then summed using Equation 14. Thus, the fuzzy decision matrix represented in Equation 31 is created.


$$\begin{array}{l} x=\left[\begin{array}{ccc} x_{11} & \cdots & x_{1n}\\ \vdots & \ddots & \vdots \\ x_{m1} & \cdots & x_{mn} \end{array}\right] \end{array}$$ (31)

Wherein *m* is the number of alternatives. Next, the elements of fuzzy decision matrix are defuzzified using Equation 32.


$$\begin{array}{l} q_{ij}=score\left(x_{ij}\right) \end{array}$$ (32)

Afterwards, the values are normalized according to max-min normalization. Next, the gray relation approach and WASPAS product values are calculated with the help of Equations 33, 34.


$$\begin{array}{lll} {𝔖}_{i} & = & \overset{n}{\underset{j=1}{\sum }}{\omega_{j}u}_{ij} \end{array}$$ (33)

$$\begin{array}{lll} {𝔓}_{i} & = & \overset{n}{\underset{j=1}{\sum }}{u_{ij}}^{\omega_{j}} \end{array}$$ (34)

Wherein u is the normalized values of q. In the next step, three strategies are computed via Equations 35–37.


$$\begin{array}{lll} {k_{a}}_{i} & = & \frac{{𝔖}_{i}+{𝔓}_{i}}{\sum_{i=1}^{m}\left({𝔖}_{i}+{𝔓}_{i}\right)} \end{array}$$ (35)

$$\begin{array}{lll} {k_{b}}_{i} & = & \frac{{𝔖}_{i}}{\min{𝔖}_{i}}+\frac{{𝔓}_{i}}{\min{𝔓}_{i}} \end{array}$$ (36)

$$\begin{array}{lll} {k_{c}}_{i} & = & \frac{T{𝔖}_{i}+\left(1-T\right){𝔓}_{i}}{T\max{𝔖}_{i}+\left(1-T\right)\max{𝔓}_{i}} \end{array}$$ (37)

Wherein T is between 0 and 1. The coefficient equals 0.5, generally. Finally, the performance scores of alternatives are obtained by Equation 38.


$$\begin{array}{l} k_{i}={\left({k_{a}}_{i}{k_{b}}_{i}{k_{c}}_{i}\right)}^{\frac{1}{3}}+\frac{1}{3}\left({k_{a}}_{i}+{k_{b}}_{i}+{k_{c}}_{i}\right) \end{array}$$ (38)

## 4 Analysis

The results of the selection of the most appropriate leadership style based on SFGAL for three various sectors are presented in this section with tables and figures.

### 4.1 Expert prioritization

Experts have varying levels of expertise. However, it's impossible to measure this directly. However, it is possible to obtain indicators related to an expertise score. Age, years of experience, and the number of certifications or patents are the variables that most clearly indicate an expert's expertise. Machine learning is used to calculate expertise scores using these variables. Within the scope of this manuscript, 10 managers with at least 25 years of experience in the human resources department are interviewed. The age, years of experience and number of certificates/patents of these 10 experts are summarized in Table 1.

**Table 1:** Ten experts' information.

Experts	Age	Experience (years)	Certificates or patents
Expert1	55	33	4
Expert2	48	26	2
Expert3	45	25	1
Expert4	58	38	6
Expert5	55	33	3
Expert6	60	39	6
Expert7	48	26	2
Expert8	56	35	4
Expert9	56	36	5
Expert10	52	32	4

As can be seen from Table 1, the average age of the experts is 53.3 years, while the expert with the maximum number of certificates/patents has six certificates/patents. Other descriptive statistics of these variables are reported in Table 2.

**Table 2:** Descriptive statistics.

Statistics	Age	Experience (years)	Certificates or patents
Mean	53.3	32.3	3.7
Min	45	25	1
Max	60	39	6
St. Dev.	4.627	4.818	1.616

The experts' years of experience range from 25 to 39. While the years and experience period are unitized with years, the other variable has the unit of amount. For this reason, the values are standardized with Equations 2–4. The standardized matrix is shared in Table 3.

**Table 3:** Standardized matrix.

Experts	Age	Experience (years)	Certificates or patents
Expert1	0.116	0.046	0.059
Expert2	−0.362	−0.414	−0.333
Expert3	−0.567	−0.479	−0.528
Expert4	0.321	0.374	0.450
Expert5	0.116	0.046	−0.137
Expert6	0.458	0.440	0.450
Expert7	−0.362	−0.414	−0.333
Expert8	0.185	0.177	0.059
Expert9	0.185	0.243	0.254
Expert10	−0.089	−0.020	0.059

Afterwards, the covariance coefficients between age, experience and number of certificates or patents are calculated via Equation 6. In other words, the common variance between variables is determined. Then, the covariance matrix is created. The covariance matrix is illustrated in Table 4.

**Table 4:** Covariance matrix.

Variables	Age	Experience (years)	Certificates or patents
Age	0.100	0.098	0.094
Experience (years)	0.098	0.100	0.096
Certificates or patents	0.094	0.096	0.100

The eigenvalues of covariance matrix are determined by solving Equation 8. The eigenvalues equal to 0.292032, 0.006754, and 0.001214. Thus, the maximum eigenvalue is 0.292032. Next, the eigenvector for maximum eigenvalue is obtained by solving Equation 9. The eigenvector is displayed in Table 5.

**Table 5:** Eigenvector.

Eigenvector
0.576941
0.582317
0.572752

**Figure 2:**
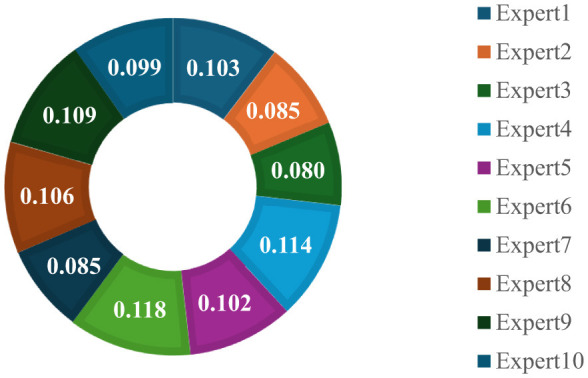
The expertise scores of experts.

Finally, the projection of data set in Table 1 is created using Equation 10. Then, the projection values are normalized with Equation 11. Thus, the expertise scores of 10 experts are illustrated in Figure 2.

According to expertise scores of experts in Figure 2, Expert 6 has the maximum expertise score with 0.118. Expert 4 has the expertise score with 0.114. These expertise scores are used in constructing decision matrices and calculating analyses.

### 4.2 Evaluation of sectors

For evaluation, the criteria set is selected as employee commitment (ECMMT), team productivity (TMPDV), innovation (INVTN), job security (JBSCR), and sustainability (SSTNBY). The selection of leadership style can be influenced by multiple performance criteria. Employee commitment (ECMMT) affects how well leaders can build trust, motivation, and loyalty among team members, which is essential for long-term organizational success. Team productivity (TMPDV) relates to the leader's ability to organize work, optimize resources, and maintain efficiency, directly impacting operational outcomes. Innovation (INVTN) reflects how leaders encourage creativity, risk-taking, and the development of new ideas, which is vital in competitive and fast-changing industries. Job security (JBSCR) influences the stability and confidence of employees, requiring leadership styles that ensure safe, fair, and predictable work environments. Sustainability (SSTNBY) involves long-term strategic thinking, environmental responsibility, and ethical practices, guiding leaders to make decisions that balance current needs with future goals.

Similarly, four types of leadership in SFGAL are Win-Win (WW), self-oriented (SO), self-neglecting/over-giving (SN), and lose-lose (LL). Self-oriented (SO) leaders show high self-goal awareness but low awareness of followers' goals, focusing more on their own vision and objectives. Self-neglecting/over-giving (SN) leaders have low self-goal awareness but high follower-goal awareness, prioritizing the needs of others even at the expense of their own professional growth. Lose-Lose (LL) leaders lack both self-goal and follower-goal awareness, resulting in poor outcomes for both the leader and the team.

Ten experts evaluate the criteria and alternatives for the energy, automotive, healthcare, and information and communication technologies sectors. Each expert has at least 25 years of professional experience and holds a senior executive position in their respective industry. All evaluations were conducted through online interviews, allowing for detailed discussions on sector-specific priorities and leadership needs. The experts' extensive experience and strategic decision-making backgrounds provide a strong basis for reliable and informed assessments in the study.

#### 4.2.1 Energy sector

**Figure 3:**
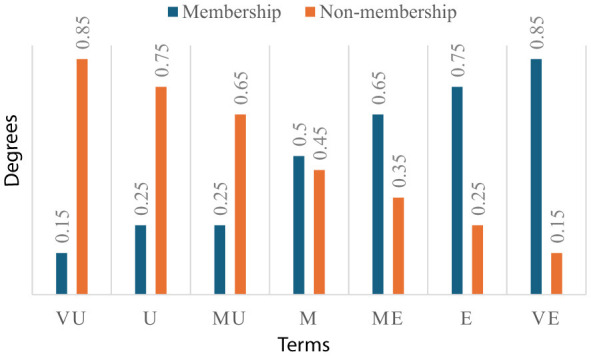
Linguistic terms.

For the energy sector, criteria evaluations from 10 experts are collected using the linguistic terms in Figure 3.

Experts' criteria assessments for the energy sector, according to the linguistic terms in Figure 3, are expressed in Table 6.

**Table 6:** Assessments (energy).

Experts	ECMMT	TMPDV	INVTN	JBSCR	SSTNBY
Expert1	U(2)	M.E.(5)	M.(4)	E.(6)	V.U.(1)
Expert2	V.E.(7)	M.(4)	U(2)	V.E.(7)	U(2)
Expert3	U(2)	E.(6)	E.(6)	M.E.(5)	V.E.(7)
Expert4	M.E.(5)	V.E.(7)	M.U.(3)	V.U.(1)	V.U.(1)
Expert5	U(2)	U(2)	V.U.(1)	V.U.(1)	M.U.(3)
Expert6	E.(6)	M.(4)	M.E.(5)	V.E.(7)	V.E.(7)
Expert7	V.E.(7)	V.E.(7)	M.U.(3)	E.(6)	E.(6)
Expert8	M.(4)	U(2)	M.(4)	E.(6)	M.U.(3)
Expert9	V.E.(7)	E.(6)	M.U.(3)	M.(4)	U(2)
Expert10	V.U.(1)	V.E.(7)	M.E.(5)	M.(4)	M.(4)

These assessments are transformed into Koch Snowflake fuzzy numbers and using Equations 22, 23, the numbers are normalized. In this manuscript, all criteria are useful. Thus, the fuzzy input data matrix and the normalized matrix are the same. The normalized matrix is exhibited in Table 7.

**Table 7:** Normalized matrix (energy).

Experts	ECMMT		TMPDV		INVTN		JBSCR		SSTNBY	
Expert1	0.250	0.750	0.650	0.350	0.500	0.450	0.750	0.250	0.150	0.850
Expert2	0.850	0.150	0.500	0.450	0.250	0.750	0.850	0.150	0.250	0.750
Expert3	0.250	0.750	0.750	0.250	0.750	0.250	0.650	0.350	0.850	0.150
Expert4	0.650	0.350	0.850	0.150	0.250	0.650	0.150	0.850	0.150	0.850
Expert5	0.250	0.750	0.250	0.750	0.150	0.850	0.150	0.850	0.250	0.650
Expert6	0.750	0.250	0.500	0.450	0.650	0.350	0.850	0.150	0.850	0.150
Expert7	0.850	0.150	0.850	0.150	0.250	0.650	0.750	0.250	0.750	0.250
Expert8	0.500	0.450	0.250	0.750	0.500	0.450	0.750	0.250	0.250	0.650
Expert9	0.850	0.150	0.750	0.250	0.250	0.650	0.500	0.450	0.250	0.750
Expert10	0.150	0.850	0.850	0.150	0.650	0.350	0.500	0.450	0.500	0.450

Afterwards, the normalized numbers are multiplied by the expertise scores in Figure 2 using Equation 24. Next, the defuzzified numbers are obtained via Equation 25. For this, score function is used. The defuzzified matrix is presented in Table 8.

**Table 8:** Defuzzified matrix (energy).

Experts	ECMMT	TMPDV	INVTN	JBSCR	SSTNBY
Expert1	−0.944	−0.787	−0.847	−0.720	−0.969
Expert2	−0.682	−0.873	−0.953	−0.682	−0.953
Expert3	−0.956	−0.780	−0.780	−0.833	−0.701
Expert4	−0.765	−0.586	−0.918	−0.966	−0.966
Expert5	−0.944	−0.944	−0.970	−0.970	−0.927
Expert6	−0.684	−0.827	−0.759	−0.575	−0.575
Expert7	−0.682	−0.682	−0.939	−0.765	−0.765
Expert8	−0.843	−0.942	−0.843	−0.711	−0.924
Expert9	−0.604	−0.706	−0.922	−0.839	−0.941
Expert10	−0.971	−0.637	−0.796	−0.854	−0.854

Using the defuzzified numbers, the maximum and minimum numbers for each criterion are selected according to Equations 26, 27. Then, the difference between these numbers is calculated by Equation 28. Later, the weights of criteria are computed via Equation 29. The results are summarized in Table 9.

**Table 9:** Weights of criteria (energy).

Parameters	ECMMT	TMPDV	INVTN	JBSCR	SSTNBY
Max	−0.604	−0.586	−0.759	−0.575	−0.575
Min	−0.971	−0.944	−0.970	−0.970	−0.969
Diff	0.367	0.359	0.211	0.395	0.394
Weight	0.213	0.208	0.122	0.229	0.229

According to weights in Table 9, the most important criteria are job security and sustainability with 0.229 and 0.229, respectively. The reliability of weights is tested with second assessments. Using Equation 30, RI value is calculated. For energy sector, the RI is 0.04. In other words, since this value is smaller than 0.1, the result is reliable.

In the next step of fuzzy decision model, these 10 experts evaluate the four types of leadership regarding to criteria using linguistic terms in Figure 3. The evaluations are shown in Table 10.

**Table 10:** Evaluations (energy).

Alter- natives	ECMMT	TMPDV	INVTN	JBSCR	SSTNBY
WW	V.E.(7)	M.E.(5)	V.E.(7)	V.E.(7)	M.E.(5)
SO	E.(6)	M.E.(5)	E.(6)	M.E.(5)	M.E.(5)
SN	V.E.(7)	V.E.(7)	U(2)	V.E.(7)	M.E.(5)
LL	M.(4)	E.(6)	M.(4)	V.U.(1)	U(2)
WW	M.E.(5)	V.E.(7)	E.(6)	M.E.(5)	E.(6)
SO	M.E.(5)	E.(6)	M.E.(5)	E.(6)	M.E.(5)
SN	V.E.(7)	M.(4)	U(2)	M.E.(5)	M.E.(5)
LL	M.U.(3)	V.U.(1)	V.E.(7)	E.(6)	V.E.(7)
WW	V.E.(7)	V.E.(7)	M.E.(5)	V.E.(7)	M.E.(5)
SO	M.E.(5)	E.(6)	E.(6)	E.(6)	E.(6)
SN	M.U.(3)	V.U.(1)	V.E.(7)	V.U.(1)	E.(6)
LL	M.E.(5)	V.E.(7)	M.(4)	V.U.(1)	M.(4)
WW	E.(6)	M.E.(5)	E.(6)	E.(6)	E.(6)
SO	M.E.(5)	E.(6)	M.E.(5)	M.E.(5)	E.(6)
SN	M.U.(3)	V.U.(1)	M.U.(3)	U(2)	M.U.(3)
LL	U(2)	V.E.(7)	U(2)	E.(6)	E.(6)
WW	E.(6)	E.(6)	V.E.(7)	E.(6)	E.(6)
SO	E.(6)	E.(6)	M.E.(5)	E.(6)	M.E.(5)
SN	V.E.(7)	V.E.(7)	V.E.(7)	M.E.(5)	E.(6)
LL	V.E.(7)	V.U.(1)	E.(6)	M.E.(5)	E.(6)
WW	E.(6)	M.E.(5)	V.E.(7)	M.E.(5)	M.E.(5)
SO	M.E.(5)	E.(6)	E.(6)	M.E.(5)	E.(6)
SN	M.U.(3)	M.U.(3)	M.E.(5)	U(2)	V.E.(7)
LL	V.E.(7)	M.U.(3)	V.E.(7)	M.(4)	M.U.(3)
WW	M.E.(5)	E.(6)	M.E.(5)	E.(6)	E.(6)
SO	E.(6)	M.E.(5)	M.E.(5)	M.E.(5)	E.(6)
SN	M.E.(5)	M.E.(5)	M.U.(3)	M.E.(5)	M.(4)
LL	M.E.(5)	E.(6)	E.(6)	M.E.(5)	U(2)
WW	M.E.(5)	M.E.(5)	V.E.(7)	E.(6)	V.E.(7)
SO	E.(6)	M.E.(5)	M.E.(5)	E.(6)	E.(6)
SN	V.E.(7)	U(2)	U(2)	U(2)	M.U.(3)
LL	V.E.(7)	U(2)	V.U.(1)	M.U.(3)	V.U.(1)
WW	V.E.(7)	V.E.(7)	V.E.(7)	E.(6)	V.E.(7)
SO	M.E.(5)	E.(6)	E.(6)	M.E.(5)	E.(6)
SN	M.U.(3)	E.(6)	M.U.(3)	M.U.(3)	U(2)
LL	M.(4)	U(2)	V.U.(1)	V.U.(1)	V.U.(1)
WW	V.E.(7)	E.(6)	M.E.(5)	V.E.(7)	V.E.(7)
SO	E.(6)	M.E.(5)	M.E.(5)	M.E.(5)	M.E.(5)
SN	M.E.(5)	E.(6)	M.(4)	M.E.(5)	M.E.(5)
LL	U(2)	V.U.(1)	M.E.(5)	V.U.(1)	E.(6)

The evaluations are transformed into Koch Snowflake fuzzy numbers regarding Figure 3. Then, Equations 14, 16 are used respectively. Thus, the fuzzy decision matrix is constructed. The fuzzy decision matrix is given in Table 11.

**Table 11:** Fuzzy decision matrix (energy).

Alternatives	ECMMT		TMPDV		INVTN		JBSCR		SSTNBY	
WW	0.776	0.225	0.748	0.252	0.793	0.208	0.769	0.232	0.765	0.236
SO	0.704	0.296	0.715	0.285	0.695	0.305	0.692	0.309	0.715	0.285
SN	0.664	0.324	0.612	0.388	0.520	0.466	0.526	0.476	0.612	0.379
LL	0.648	0.347	0.553	0.455	0.619	0.383	0.489	0.509	0.552	0.449

Afterwards, the values are defuzzified via Equation 32. For this, the score function is used. Then, the defuzzified values are normalized according to max-min normalization. The max-min normalization is displayed in Table 12.

**Table 12:** Max–min normalization (energy).

Alter-natives	ECMMT	TMPDV	INVTN	JBSCR	SSTNBY
WW	1.000	1.000	1.000	1.000	1.000
SO	0.431	0.834	0.638	0.727	0.771
SN	0.155	0.319	0.000	0.126	0.305
LL	0.000	0.000	0.348	0.000	0.000

Next, the gray relation approach and WASPAS product values are calculated with the help of Equations 33, 34. Later, three strategies are computed via Equations 35–37. Finally, the performance scores of four types of leadership are obtained by Equation 38. The results are presented in Table 13.

**Table 13:** Performance scores (energy).

Alter-natives	S	P	Ka	Kb	Kc	K
WW	1.000	5.000	0.393	29.165	1.000	12.440
SO	0.686	4.618	0.347	21.347	0.884	9.398
SN	0.198	2.846	0.199	7.878	0.507	3.789
LL	0.043	0.879	0.060	2.000	0.154	1.003

According to *K* values in Table 13, the optimal leadership types are win-win and self-oriented with 12.44 and 9.398, respectively for energy sector. The high score of the win-win type indicates that leaders who balance their own goals with the goals of their followers can best address the sector's priorities, such as occupational safety and sustainability. The self-oriented type, ranking second, suggests that a strong focus on the leader's own vision and strategic objectives can also be effective, particularly in guiding long-term energy investments and innovation. These results highlight the importance of combining collaborative and visionary leadership approaches in the energy sector.

#### 4.2.2 Automotive sector

The same experts' criteria assessments for the automative sector, according to the linguistic terms in Figure 3, are expressed in Table 14.

**Table 14:** Assessments (automative).

Experts	ECMMT	TMPDV	INVTN	JBSCR	SSTNBY
Expert1	E.(6)	M.U.(3)	U(2)	V.E.(7)	M.E.(5)
Expert2	E.(6)	V.E.(7)	V.E.(7)	M.(4)	V.E.(7)
Expert3	U(2)	U(2)	M.(4)	V.E.(7)	M.E.(5)
Expert4	E.(6)	U(2)	M.U.(3)	M.E.(5)	U(2)
Expert5	E.(6)	M.E.(5)	M.U.(3)	M.U.(3)	M.(4)
Expert6	V.U.(1)	E.(6)	M.E.(5)	M.U.(3)	U(2)
Expert7	M.(4)	M.U.(3)	V.U.(1)	M.E.(5)	M.U.(3)
Expert8	U(2)	M.(4)	V.E.(7)	M.E.(5)	U(2)
Expert9	V.U.(1)	M.E.(5)	V.E.(7)	E.(6)	U(2)
Expert10	E.(6)	V.E.(7)	E.(6)	M.U.(3)	U(2)

**Figure 4:**
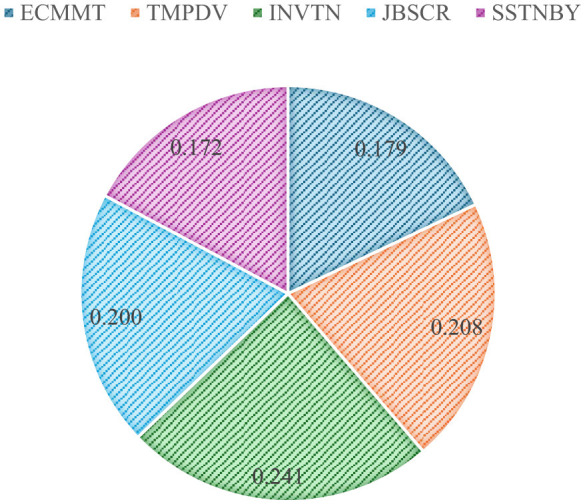
Weights of criteria (automotive).

The analysis process detailed for the energy sector is applied to this sector as well. The criteria weightings, according to CIMAS, are visualized in Figure 4.

According to weights in Figure 4, the most important criteria are innovation and team productivity with 0.241 and 0.208, respectively. RI is 0.02. In next step, CoCoSo is applied. The evaluation of 10 experts is shown in Table 15.

**Table 15:** Evaluation (automative).

Alter- natives	ECMMT	TMPDV	INVTN	JBSCR	SSTNBY
WW	M.E.(5)	E.(6)	E.(6)	V.E.(7)	V.E.(7)
SO	M.E.(5)	E.(6)	M.E.(5)	M.(4)	V.E.(7)
SN	U(2)	V.E.(7)	U(2)	V.E.(7)	E.(6)
LL	U(2)	E.(6)	M.E.(5)	M.E.(5)	E.(6)
WW	M.E.(5)	V.E.(7)	E.(6)	V.E.(7)	M.E.(5)
SO	M.E.(5)	V.E.(7)	E.(6)	M.(4)	M.(4)
SN	V.U.(1)	V.U.(1)	E.(6)	M.(4)	U(2)
LL	V.E.(7)	M.E.(5)	V.U.(1)	M.U.(3)	U(2)
WW	M.E.(5)	M.E.(5)	E.(6)	M.E.(5)	V.E.(7)
SO	V.E.(7)	V.E.(7)	V.E.(7)	M.E.(5)	M.(4)
SN	M.U.(3)	E.(6)	M.(4)	E.(6)	U(2)
LL	M.(4)	E.(6)	V.U.(1)	M.U.(3)	M.(4)
WW	V.E.(7)	M.E.(5)	M.E.(5)	E.(6)	E.(6)
SO	M.E.(5)	V.E.(7)	M.E.(5)	E.(6)	E.(6)
SN	M.E.(5)	M.(4)	U(2)	U(2)	V.E.(7)
LL	M.U.(3)	V.E.(7)	M.(4)	M.E.(5)	M.E.(5)
WW	V.E.(7)	M.E.(5)	E.(6)	M.E.(5)	V.E.(7)
SO	M.(4)	M.E.(5)	V.E.(7)	M.(4)	M.(4)
SN	M.(4)	E.(6)	V.E.(7)	V.U.(1)	U(2)
LL	M.E.(5)	M.U.(3)	M.U.(3)	V.U.(1)	U(2)
WW	V.E.(7)	V.E.(7)	V.E.(7)	E.(6)	V.E.(7)
SO	M.E.(5)	M.(4)	V.E.(7)	V.E.(7)	M.(4)
SN	M.U.(3)	U(2)	E.(6)	E.(6)	E.(6)
LL	M.(4)	M.E.(5)	V.E.(7)	U(2)	E.(6)
WW	M.E.(5)	V.E.(7)	M.E.(5)	E.(6)	V.E.(7)
SO	V.E.(7)	V.E.(7)	M.E.(5)	E.(6)	M.E.(5)
SN	M.U.(3)	V.U.(1)	M.U.(3)	M.E.(5)	V.U.(1)
LL	V.U.(1)	E.(6)	U(2)	V.E.(7)	V.U.(1)
WW	E.(6)	V.E.(7)	V.E.(7)	M.E.(5)	V.E.(7)
SO	E.(6)	V.E.(7)	M.(4)	E.(6)	E.(6)
SN	M.U.(3)	M.(4)	U(2)	U(2)	M.(4)
LL	V.E.(7)	M.E.(5)	V.E.(7)	M.E.(5)	E.(6)
WW	V.E.(7)	E.(6)	V.E.(7)	M.E.(5)	E.(6)
SO	M.E.(5)	M.E.(5)	M.E.(5)	E.(6)	M.E.(5)
SN	V.E.(7)	V.U.(1)	M.U.(3)	V.E.(7)	U(2)
LL	E.(6)	E.(6)	E.(6)	E.(6)	U(2)
WW	V.E.(7)	E.(6)	E.(6)	M.E.(5)	V.E.(7)
SO	M.E.(5)	V.E.(7)	M.(4)	V.E.(7)	M.(4)
SN	V.U.(1)	V.E.(7)	M.(4)	V.E.(7)	E.(6)
LL	M.(4)	M.U.(3)	E.(6)	V.E.(7)	V.E.(7)

**Figure 5:**
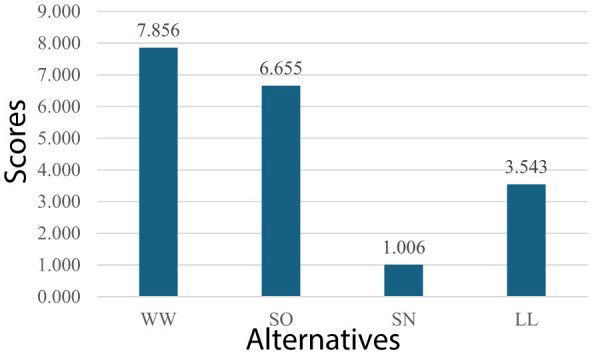
Performance scores (automotive).

Using these evaluations, the performance scores are obtained. The performance scores of four types of leadership for automative sector are presented in Figure 5.

As can be seen values in Figure 5, the most optimal types of leadership for automotive are win-win and self-oriented, respectively. The dominance of the win-win type shows that a balanced focus on both leader and follower goals is essential to support the sector's key priorities, such as innovation and team productivity. The self-oriented type ranking second indicates that a leader's strong personal vision and strategic direction can drive technological advancements, such as electric and autonomous vehicle development. These findings suggest that combining collaborative engagement with visionary leadership can yield the best results in the automotive industry.

#### 4.2.3 Health sector

The same experts' criteria assessments for the health sector, according to the linguistic terms in Figure 3, are expressed in Table 16.

**Table 16:** Assessments (health).

Experts	ECMMT	TMPDV	INVTN	JBSCR	SSTNBY
Expert1	M.U.(3)	E.(6)	M.E.(5)	V.E.(7)	E.(6)
Expert2	M.(4)	E.(6)	U(2)	V.E.(7)	M.(4)
Expert3	V.U.(1)	E.(6)	M.E.(5)	M.(4)	M.E.(5)
Expert4	M.E.(5)	U(2)	M.E.(5)	U(2)	M.E.(5)
Expert5	M.U.(3)	V.U.(1)	U(2)	M.E.(5)	M.(4)
Expert6	E.(6)	M.(4)	V.U.(1)	M.(4)	M.U.(3)
Expert7	M.U.(3)	M.(4)	M.(4)	V.U.(1)	M.E.(5)
Expert8	M.E.(5)	M.(4)	M.(4)	M.(4)	M.E.(5)
Expert9	V.E.(7)	M.U.(3)	M.U.(3)	M.E.(5)	M.E.(5)
Expert10	M.E.(5)	E.(6)	V.U.(1)	M.E.(5)	M.(4)

**Figure 6:**
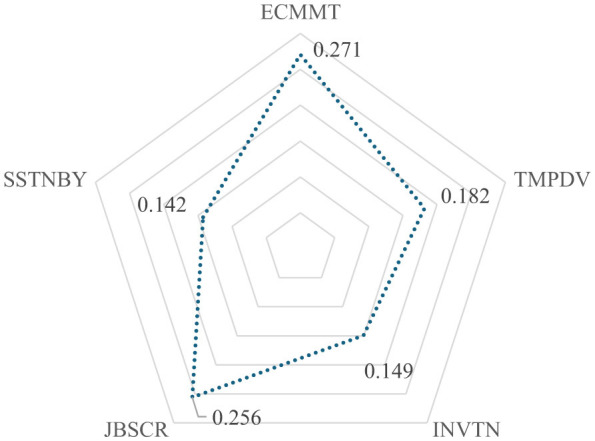
Weights of criteria (health).

The analysis process detailed for the energy sector is applied to this sector as well. The criteria weightings, according to CIMAS, are visualized in Figure 6.

According to weights in Figure 6, the most important criteria for health sector are employee commitment and job security with 0.271 and 0.256, respectively. RI equals 0.08. In next step, CoCoSo is applied. The evaluation of 10 experts is shown in Table 17.

**Table 17:** Evaluation (health).

Alter- natives	ECMMT	TMPDV	INVTN	JBSCR	SSTNBY
WW	M.E.(5)	V.E.(7)	E.(6)	M.E.(5)	V.E.(7)
SO	M.E.(5)	U(2)	M.E.(5)	M.(4)	V.U.(1)
SN	M.(4)	M.E.(5)	M.(4)	M.E.(5)	E.(6)
LL	V.E.(7)	E.(6)	M.U.(3)	M.U.(3)	M.E.(5)
WW	E.(6)	V.E.(7)	E.(6)	M.E.(5)	V.E.(7)
SO	E.(6)	V.U.(1)	V.E.(7)	V.E.(7)	E.(6)
SN	E.(6)	M.(4)	M.E.(5)	M.(4)	E.(6)
LL	U(2)	V.U.(1)	V.E.(7)	M.U.(3)	V.E.(7)
WW	V.E.(7)	V.E.(7)	V.E.(7)	E.(6)	V.E.(7)
SO	V.E.(7)	U(2)	M.U.(3)	M.(4)	M.E.(5)
SN	E.(6)	E.(6)	E.(6)	E.(6)	M.(4)
LL	U(2)	E.(6)	V.U.(1)	V.U.(1)	E.(6)
WW	V.E.(7)	M.E.(5)	V.E.(7)	E.(6)	V.E.(7)
SO	V.E.(7)	M.U.(3)	V.E.(7)	M.U.(3)	E.(6)
SN	M.(4)	M.E.(5)	E.(6)	M.(4)	M.E.(5)
LL	E.(6)	V.E.(7)	E.(6)	U(2)	U(2)
WW	E.(6)	E.(6)	M.E.(5)	M.E.(5)	V.E.(7)
SO	M.(4)	M.(4)	V.E.(7)	E.(6)	M.(4)
SN	E.(6)	M.E.(5)	E.(6)	M.E.(5)	M.E.(5)
LL	M.E.(5)	M.E.(5)	M.(4)	M.U.(3)	M.E.(5)
WW	V.E.(7)	E.(6)	V.E.(7)	E.(6)	V.E.(7)
SO	V.U.(1)	V.E.(7)	M.E.(5)	V.U.(1)	M.(4)
SN	M.E.(5)	E.(6)	M.E.(5)	M.E.(5)	M.E.(5)
LL	M.U.(3)	M.U.(3)	V.U.(1)	M.E.(5)	M.U.(3)
WW	V.E.(7)	V.E.(7)	E.(6)	M.E.(5)	V.E.(7)
SO	U(2)	U(2)	M.U.(3)	V.U.(1)	M.(4)
SN	M.(4)	E.(6)	M.E.(5)	M.(4)	E.(6)
LL	V.U.(1)	E.(6)	M.(4)	U(2)	M.E.(5)
WW	V.E.(7)	V.E.(7)	M.E.(5)	E.(6)	M.E.(5)
SO	M.U.(3)	M.E.(5)	M.E.(5)	V.U.(1)	M.E.(5)
SN	M.E.(5)	M.E.(5)	E.(6)	E.(6)	E.(6)
LL	U(2)	M.U.(3)	V.U.(1)	M.E.(5)	M.E.(5)
WW	E.(6)	V.E.(7)	V.E.(7)	E.(6)	E.(6)
SO	E.(6)	E.(6)	V.U.(1)	E.(6)	V.U.(1)
SN	E.(6)	M.(4)	E.(6)	M.E.(5)	E.(6)
LL	U(2)	E.(6)	V.E.(7)	V.E.(7)	E.(6)
WW	E.(6)	V.E.(7)	V.E.(7)	V.E.(7)	E.(6)
SO	V.U.(1)	E.(6)	E.(6)	M.U.(3)	M.(4)
SN	M.(4)	E.(6)	M.(4)	M.E.(5)	M.(4)
LL	M.E.(5)	U(2)	U(2)	E.(6)	U(2)

**Figure 7:**
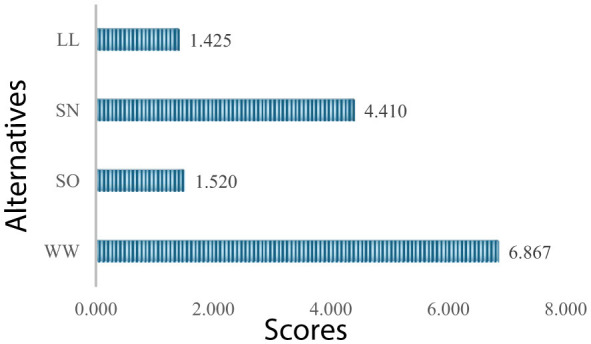
Performance scores (health).

Using these evaluations, the performance scores are obtained. The performance scores of four types of leadership for health sector are presented in Figure 7.

As can be seen values in Figure 7, the most optimal types of leadership for health sector are win-win and self-neglecting/over-giving, respectively. The win-win type's leading position indicates that balancing the leader's own goals with the needs of healthcare staff is vital for improving both employee commitment and patient safety. The self-neglecting/over-giving type ranking second shows that, in high-pressure situations such as emergencies, leaders who prioritize the needs of their team over their own professional goals can be highly effective. These results highlight the importance of combining balanced, mutual-gain leadership with self-sacrificing approaches in the healthcare sector.

#### 4.2.4 Information and communication technologies sector

These experts' criteria assessments for the information and communication technologies sector, according to the linguistic terms in Figure 3, are expressed in Table 18.

**Table 18:** Assessments (information and communication technologies).

Experts	ECMMT	TMPDV	INVTN	JBSCR	SSTNBY
Expert1	V.E.(7)	V.U.(1)	V.E.(7)	E.(6)	V.U.(1)
Expert2	M.E.(5)	U(2)	M.(4)	E.(6)	V.U.(1)
Expert3	E.(6)	V.E.(7)	E.(6)	M.E.(5)	V.E.(7)
Expert4	V.U.(1)	M.U.(3)	M.U.(3)	E.(6)	M.U.(3)
Expert5	V.E.(7)	V.U.(1)	V.E.(7)	U(2)	E.(6)
Expert6	M.(4)	V.U.(1)	M.E.(5)	U(2)	U(2)
Expert7	E.(6)	M.U.(3)	V.U.(1)	V.E.(7)	U(2)
Expert8	M.E.(5)	M.E.(5)	E.(6)	M.U.(3)	E.(6)
Expert9	M.(4)	M.U.(3)	V.E.(7)	M.U.(3)	V.U.(1)
Expert10	M.(4)	E.(6)	M.(4)	U(2)	M.E.(5)

**Figure 8:**
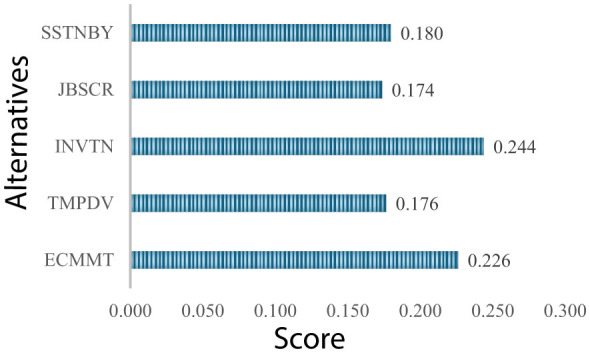
Weights of criteria (information and communication technologies).

The analysis process detailed for the energy sector is applied to this sector as well. The criteria weightings, according to CIMAS, are visualized in Figure 8.

According to weights in Figure 8, the most important criteria for this sector are innovation and employee commitment with 0.244 and 0.226, respectively. RI equals 0.04. In next step, CoCoSo is applied. The evaluation of 10 experts is shown in Table 19.

**Table 19:** Evaluation (information and communication technologies).

Alter- natives	ECMMT	TMPDV	INVTN	JBSCR	SSTNBY
WW	V.E.(7)	E.(6)	E.(6)	V.E.(7)	V.E.(7)
SO	E.(6)	V.E.(7)	E.(6)	V.E.(7)	V.E.(7)
SN	M.E.(5)	U(2)	E.(6)	U(2)	V.U.(1)
LL	M.E.(5)	M.E.(5)	U(2)	M.U.(3)	M.E.(5)
WW	V.E.(7)	E.(6)	V.E.(7)	E.(6)	V.E.(7)
SO	V.E.(7)	E.(6)	E.(6)	V.E.(7)	E.(6)
SN	U(2)	M.E.(5)	E.(6)	V.E.(7)	V.U.(1)
LL	M.E.(5)	E.(6)	U(2)	M.(4)	M.U.(3)
WW	V.E.(7)	E.(6)	E.(6)	V.E.(7)	E.(6)
SO	E.(6)	E.(6)	M.E.(5)	E.(6)	V.E.(7)
SN	M.U.(3)	E.(6)	V.E.(7)	M.E.(5)	V.E.(7)
LL	V.E.(7)	M.U.(3)	V.U.(1)	M.U.(3)	E.(6)
WW	V.E.(7)	V.E.(7)	E.(6)	E.(6)	E.(6)
SO	V.E.(7)	E.(6)	V.E.(7)	E.(6)	V.E.(7)
SN	U(2)	V.E.(7)	V.U.(1)	V.E.(7)	M.U.(3)
LL	M.(4)	M.U.(3)	M.(4)	E.(6)	M.U.(3)
WW	V.E.(7)	V.E.(7)	E.(6)	V.E.(7)	V.E.(7)
SO	M.E.(5)	E.(6)	V.E.(7)	V.E.(7)	V.E.(7)
SN	V.E.(7)	E.(6)	M.U.(3)	M.E.(5)	V.U.(1)
LL	V.U.(1)	M.U.(3)	V.U.(1)	U(2)	U(2)
WW	V.E.(7)	V.E.(7)	V.E.(7)	E.(6)	V.E.(7)
SO	M.E.(5)	M.E.(5)	M.E.(5)	E.(6)	M.E.(5)
SN	E.(6)	E.(6)	E.(6)	U(2)	V.U.(1)
LL	M.(4)	U(2)	M.E.(5)	M.(4)	M.U.(3)
WW	V.E.(7)	V.E.(7)	V.E.(7)	E.(6)	E.(6)
SO	E.(6)	E.(6)	E.(6)	M.E.(5)	V.E.(7)
SN	M.U.(3)	V.U.(1)	V.E.(7)	M.(4)	M.(4)
LL	V.E.(7)	V.E.(7)	E.(6)	V.U.(1)	E.(6)
WW	E.(6)	V.E.(7)	E.(6)	E.(6)	E.(6)
SO	M.E.(5)	M.E.(5)	M.E.(5)	V.E.(7)	M.E.(5)
SN	V.U.(1)	V.U.(1)	M.E.(5)	V.E.(7)	V.U.(1)
LL	M.E.(5)	M.U.(3)	V.E.(7)	U(2)	M.E.(5)
WW	E.(6)	E.(6)	E.(6)	E.(6)	V.E.(7)
SO	E.(6)	E.(6)	V.E.(7)	V.E.(7)	E.(6)
SN	M.E.(5)	M.E.(5)	M.(4)	M.E.(5)	V.U.(1)
LL	M.(4)	U(2)	U(2)	E.(6)	U(2)
WW	E.(6)	E.(6)	E.(6)	V.E.(7)	V.E.(7)
SO	V.E.(7)	M.E.(5)	V.E.(7)	M.E.(5)	V.E.(7)
SN	M.(4)	V.U.(1)	U(2)	M.(4)	M.(4)
LL	V.E.(7)	M.(4)	M.E.(5)	V.U.(1)	V.U.(1)

**Figure 9:**
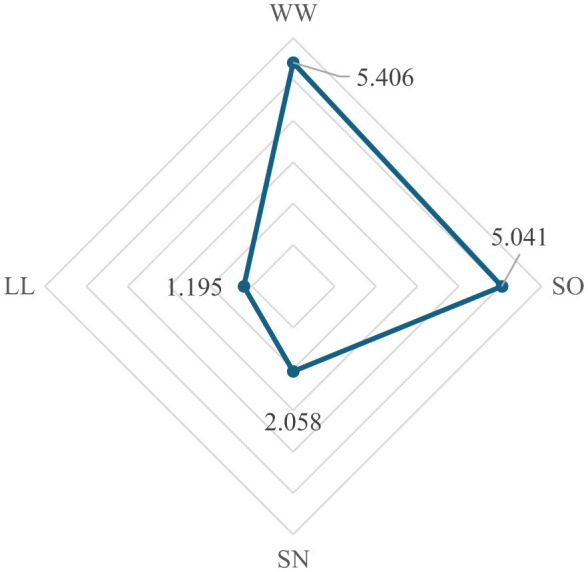
Performance scores (information and communication technologies).

Using these evaluations, the performance scores are obtained. The performance scores of four types of leadership for this sector are presented in Figure 9.

As can be seen values in Figure 9, the most optimal types of leadership for this sector are win-win and self-oriented, respectively. The strong performance of the win-win type reflects the importance of balancing leader and follower goals to foster innovation and maintain high employee engagement. They are critical in the information and communication technologies sector. The self-oriented type ranking second indicates that leaders with a strong personal vision can effectively drive technological advancements and strategic product development.

### 4.3 Sensitivity and comparative analysis

Both comparative and sensitivity analyses are performed to ensure the robustness and reliability of the results. Within the scope of sensitivity analysis, scenarios are constructed by randomly updating the criterion weight values and the priority values of the experts using Monte Carlo simulation. Thus, the effect of minimal changes in the input variables on the ranking results is examined. The ranking results of leadership types according to the seven scenarios created within this scope are summarized in Table 20.

**Table 20:** Scenario analysis of methodology.

Sectors	Scenario1	Scenario2	Scenario3	Scenario4	Scenario5	Scenario6	Scenario7
Energy							
WW	1	1	1	1	1	1	1
SO	2	2	2	2	2	2	2
SN	3	3	3	3	3	3	3
LL	4	4	4	4	4	4	4
Automotive							
WW	1	1	1	1	1	1	1
SO	2	2	2	2	2	2	2
SN	4	4	4	4	4	4	4
LL	3	3	3	3	3	3	3
Health							
WW	1	1	1	1	1	1	1
SO	3	3	3	3	3	3	3
SN	2	2	2	2	2	2	2
LL	4	4	4	4	4	4	4
ICT							
WW	1	1	1	1	1	1	1
SO	2	2	2	2	2	2	2
SN	3	3	3	3	3	3	3
LL	4	4	4	4	4	4	4

When scenario analyses are examined, the ranking of leadership types across sectors remains the same. This means that the priority of leadership types does not change even when inputs change. In addition, the results are compared using different ranking models such as TOPSIS, RAM, ARAS, and WISP. Ranking models' results and scenario analyses for ranking models are presented in Table 21.

**Table 21:** Scenario analysis of ranking models.

Sectors	TOPSIS	Scenario1	Scenario2	Scenario3	Scenario4	Scenario5	Scenario6	Scenario7		RAM	Scenario1	Scenario2	Scenario3	Scenario4	Scenario5	Scenario6	Scenario7
Energy																	
WW	1	1	1	1	2	1	1	1	WW	1	1	1	1	2	1	1	1
SO	2	3	2	2	1	2	4	2	SO	2	3	3	2	1	2	4	2
SN	3	2	3	4	3	3	3	3	SN	3	2	2	4	3	3	3	3
LL	4	4	4	3	4	4	2	4	LL	4	4	4	3	4	4	2	4
Automotive																	
WW	1	1	1	1	1	1	1	2	WW	1	1	1	1	1	1	1	2
SO	2	2	4	2	3	2	2	1	SO	2	2	4	3	3	2	2	1
SN	4	4	2	3	2	4	4	4	SN	4	4	2	2	2	4	4	4
LL	3	3	3	4	4	3	3	3	LL	3	3	3	4	4	3	3	3
Health																	
WW	1	1	1	1	1	1	3	1	WW	1	1	1	1	1	1	3	1
SO	3	3	4	3	3	3	1	3	SO	3	3	4	3	2	3	1	3
SN	2	2	2	2	2	2	2	4	SN	2	2	2	2	3	2	2	4
LL	4	4	3	4	4	4	4	2	LL	4	4	3	4	4	4	4	2
ICT																	
WW	1	1	1	1	1	2	3	1	WW	1	1	1	1	1	2	3	1
SO	2	4	2	2	2	1	2	4	SO	2	2	2	2	2	1	1	4
SN	3	2	4	3	3	3	1	3	SN	3	4	4	3	3	3	2	3
LL	4	3	3	4	4	4	4	2	LL	4	3	3	4	4	4	4	2
Sectors	ARAS	Scenario1	Scenario2	Scenario3	Scenario4	Scenario5	Scenario6	Scenario7		WISP	Scenario1	Scenario2	Scenario3	Scenario4	Scenario5	Scenario6	Scenario7
Energy																	
WW	1	1	1	1	2	2	1	1	WW	1	1	3	1	2	2	1	1
SO	2	3	3	2	1	1	4	2	SO	2	3	1	2	1	1	3	2
SN	3	2	2	4	3	3	3	3	SN	3	2	2	4	3	3	4	3
LL	4	4	4	3	4	4	2	4	LL	4	4	4	3	4	4	2	4
WW	1	1	1	1	1	1	1	2	WW	1	1	1	1	3	1	1	2
SO	2	2	2	3	3	2	2	1	SO	2	2	2	3	1	2	2	1
SN	4	4	4	2	2	4	4	4	SN	4	4	4	2	2	4	4	4
LL	3	3	3	4	4	3	3	3	LL	3	3	3	4	4	3	3	3
Health																	
WW	1	1	1	1	1	1	3	1	WW	1	3	1	3	1	1	3	1
SO	3	3	4	3	3	3	1	3	SO	3	1	4	1	3	3	1	3
SN	2	2	2	2	2	2	2	4	SN	2	2	2	2	2	2	2	4
LL	4	4	3	4	4	4	4	2	LL	4	4	3	4	4	4	4	2
ICT																	
WW	1	1	1	1	1	2	3	1	WW	1	1	1	2	1	2	3	1
SO	2	2	2	2	2	1	1	4	SO	2	2	2	1	2	1	1	4
SN	3	3	4	3	3	3	2	2	SN	3	3	4	3	3	3	2	2
LL	4	4	3	4	4	4	4	3	LL	4	4	3	4	4	4	4	3

When Table 21 is examined, the ranking of leadership types by sector is the same according to both ranking models and the proposed methodology. In other words, the results are consistent and therefore reliable. However, variations are observed in the ranking results of ranking models across the scenarios. This demonstrates the robustness of the proposed methodology and shows the superiority of the manuscript.

## 5 Discussion

The findings of this study provide important insights into how leadership effectiveness is shaped by sector-specific priorities and contribute to ongoing debates in leadership research regarding the balance between leader self-goal awareness and follower-oriented behaviors. The consistent prominence of win–win leadership across all examined sectors suggests that leadership approaches emphasizing mutual gains for leaders and followers remain highly effective when multiple performance criteria must be simultaneously considered. This result is broadly consistent with relational and exchange-based leadership theories, which emphasize reciprocity, alignment of interests, and the co-creation of value in leader–follower relationships. As Gilbert-Ouimet et al. (2025) also indicate, leadership approaches that integrate concern for both leader and follower goals are particularly well-suited to achieving multidimensional organizational outcomes, such as engagement, performance, and wellbeing.

However, the present findings extend existing literature by demonstrating that the effectiveness of leadership styles is not uniform across contexts but varies systematically with sectoral demands. In the energy sector, the strong relevance of win–win leadership reflects the need to balance occupational safety, regulatory compliance, and long-term sustainability objectives. Prior research has highlighted that visionary and self-oriented leadership traits can play a constructive role in guiding strategic investments and renewable energy transitions (Zheng et al., 2025), a pattern that is supported by the relatively high performance of self-oriented leadership in this sector. Similarly, in the automotive sector, the importance of innovation and productivity aligns with studies emphasizing the role of strong leader vision and strategic focus in technology-intensive environments, particularly in the development of electric and autonomous vehicles (Cao et al., 2025). These findings reinforce contingency-based leadership perspectives, suggesting that sectoral characteristics shape which combinations of leader and follower goal awareness are most effective.

The healthcare sector presents a more nuanced pattern. While win–win leadership remains the most suitable overall style, the relatively strong performance of self-neglecting/over-giving leadership reflects the unique pressures of high-stakes and time-critical environments. In such contexts, leadership behaviors that prioritize team needs and collective functioning may become temporarily advantageous, particularly during emergencies or crises (Babu et al., 2025). This finding resonates with prior research emphasizing situational leadership adjustments in healthcare settings and challenges overly normative assumptions that consistently valorize leader self-focus. Similarly, in the information and communication technologies sector, the coexistence of win–win and self-oriented leadership underscores the importance of balancing employee engagement with strong strategic direction in innovation-driven and rapidly changing environments. As Liu et al. (2025) note, sector-specific conditions often require hybrid leadership configurations rather than rigid adherence to a single ideal type. Moreover, Zhang and Ma (2025) highlight that leadership effectiveness is closely tied to contextual expectations and pressures, particularly in high-demand or uncertain situations.

Taken together, these findings suggest that while win–win leadership offers broad adaptability and balanced benefits across sectors, other leadership styles retain situational relevance. Rather than prescribing a single optimal leadership approach, the results support a more flexible and context-sensitive understanding of leadership effectiveness. By integrating these insights with existing theory and empirical evidence, the study advances leadership research beyond dichotomous classifications of leadership styles and highlights the value of systematically evaluating leadership strategies in relation to sector-specific priorities and organizational demands.

The discussion of the findings should be interpreted in light of the integrative nature of the proposed framework. The primary contribution of this study does not stem from any single analytical technique, but from the way leadership theory and decision-support methods are jointly employed to examine leadership effectiveness as a context-dependent and multidimensional phenomenon. By making trade-offs among multiple performance criteria explicit and analytically tractable, the framework allows for a more nuanced interpretation of leadership styles across sectors. This integrative perspective enables the findings to be discussed not merely as rankings of leadership alternatives, but as theoretically meaningful patterns that reveal how different configurations of leader and follower goal awareness align with sector-specific organizational demands.

## 6 Conclusion

This study develops and applies a sector-sensitive decision-support framework to evaluate Self-and-Follower Goal-Aware Leadership (SFGAL) styles across different industries and to reveal inter-sectoral differences in leadership effectiveness. By jointly considering multiple performance criteria, the findings demonstrate that leadership effectiveness cannot be reduced to a single dominant dimension but must be understood as a multidimensional and context-dependent phenomenon. Across all examined sectors, win–win leadership consistently emerges as the most suitable style, highlighting the importance of balancing leaders' self-goal awareness with attentiveness to followers' goals. At the same time, the relative importance of performance criteria differs across sectors, with innovation being most salient in the automotive and information and communication technologies sectors, occupational safety in the energy sector, and employee commitment in the healthcare sector. These differences underscore the need for leadership approaches that are aligned with sector-specific priorities rather than universal prescriptions. The results further indicate that self-oriented leadership retains situational relevance, particularly in sectors characterized by strategic complexity and innovation intensity, suggesting that leader self-goal awareness can function as a valuable resource rather than a liability when appropriately balanced.

In addition to its theoretical aspects, SFGAL has implications for leadership assessment and development. Specifically, the model can help leaders move toward the win-win quadrant. Accordingly, self-oriented leaders can learn to attend to the followers' needs, and self-neglecting/over-giving leaders can enhance their focus on their needs. In contrast, leaders who are lose-lose can receive training to strengthen self- and follower-focused awareness, attitudes, and behaviors. In sum, the SFGAL provides explanatory insight into the dynamics of leader-follower relations, as it can identify how much the leader and the followers gain from the leader's leadership practice. At the same time, it provides actionable insights directed toward improving leader-follower relations when needed. Intervention in the case of self-neglecting/over-giving leadership can target increasing leader agency, assisting the leader in setting self-goals, enhancing wellbeing, and raising awareness to balance attention to self and follower goals. In contrast, self-oriented leadership intervention can help leaders improve their empathy and listening skills, and highlight the benefits of follower development, such as improved team performance and satisfaction. Finally, lose-lose leadership would benefit from both interventions to enhance self- and follower-focused goal awareness. The SFGAL model's ultimate goal is to help leaders establish win-win relationships with their followers.

From a theoretical perspective, this study contributes to leadership research by explicitly integrating leaders' self-goal awareness into the evaluation of leadership effectiveness, thereby addressing an important imbalance in the existing literature. Methodologically, it advances leadership assessment by demonstrating the applicability of a structured, multi-criteria decision-making framework that incorporates uncertainty modeling and qualification-based expert weighting. Practically, the findings offer guidance for leadership development and policy design by emphasizing that effective leadership strategies should be tailored to sectoral demands and performance priorities. Despite these contributions, the study has limitations. The reliance on expert judgments, although systematically weighted, may still introduce subjectivity. In addition, the analysis is limited to four sectors, which may constrain the generalizability of the findings, and the selected criteria do not capture all possible dimensions of leadership effectiveness. Future research could extend the proposed framework to additional sectors, incorporate a broader set of evaluation criteria such as financial performance and digital transformation capability, and combine expert-based assessments with organizational-level empirical data. Such extensions would further strengthen the robustness and applicability of the SFGAL-based decision-support approach.

## Data Availability

The original contributions presented in the study are included in the article/supplementary material, further inquiries can be directed to the corresponding author.
